# Yeast One-Hybrid Screening to Identify Transcription Factors for *IbMYB1-4* in the Purple-Fleshed Sweet Potato (*Ipomoea batatas* [L.] Lam.)

**DOI:** 10.3390/cimb45070364

**Published:** 2023-07-12

**Authors:** Danwen Fu, Shaohua Yang, Rui Liu, Feng Gao

**Affiliations:** 1 Guangdong Provincial Key Lab of Biotechnology for Plant Development, School of Life Sciences, South China Normal University, Guangzhou 510631, China; danwenfu@yeah.net (D.F.); 15914375650@163.com (S.Y.); 2 Institute of Nanfan & Seed Industry, Guangdong Academy of Sciences, Guangzhou 510316, China; liurui701@126.com

**Keywords:** anthocyanin biosynthesis, IbERF1, IbPDC, IbPGP19, upstream transcription factors

## Abstract

IbMYB1 is a transcription factor involved in the biosynthesis of anthocyanin in the purple-fleshed sweet potato. So far, few studies have investigated transcription factors that are upstream of the promoter *IbMYB1-4*. In this study, a yeast one-hybrid screening aimed at identifying transcription factors upstream of the promoter *IbMYB1-4* was performed in the storage roots of the purple-fleshed sweet potato, and IbPDC, IbERF1, and IbPGP19 were identified as upstream binding proteins for the promoter *IbMYB1-4*. A dual luciferase reporter assay, and yeast one-hybrid assays, were employed to confirm the interaction of these binding proteins with promoters. IbERF1 was found to be an upstream transcription factor for the promoter *IbMYB1*, and is implicated in the biosynthesis of anthocyanin in the purple-fleshed sweet potato. IbERF1 plays a major role in the biosynthesis of anthocyanin in the purple-fleshed sweet potato.

## 1. Introduction

The sweet potato is a major food crop in China, and is mainly grown in tropical and subtropical regions. High levels of anthocyanin with specific physiological activity and functions are present in the purple-fleshed sweet potato (PFSP). Anthocyanin has anticancer and antioxidation functions, and plays a role in lowering blood pressure and preventing arteriosclerosis [1]. The accumulation of anthocyanin is regulated via the expression levels of several genes, including *CHI*, *CHS*, *DFR*, *UF3GT*, *ANS*, and *F3H*. Transcription factors (TFs) regulate the expression of these structural genes. These trans-acting factors comprise a group of DNA-binding proteins that bind specifically to cis-acting elements of eukaryotic genes, and regulate the activation or inhibition of gene transcription [2]. The biosynthesis of anthocyanin in the PFSP is regulated by the TF complex MYB–bHLH–WD40 (MBW) [3,4]. The *IbMYB1* gene expression was reported to correlate positively with that of related structural genes in anthocyanin biosynthesis. The *IbMYB1* promoter (length 2183 bp) was cut into four segments (−2183 bp~−1684 bp, −1683 bp~−1194 bp, −1193 bp~−694 bp, −693 bp~−1 bp). A previous study investigated the *IbMYB1-1* promoter for upstream TFs [5]. It was noted that high concentrations of AbA did not suppress the Leu leakiness with the promoter *IbMYB1-2* and the promoter *IbMYB1-3*, with 400 ng/mL being the minimum inhibitory AbA concentration for the PAbAi-*PIbMYB1-4* strains. However, little is known regarding TFs that are upstream of *PIbMYB1-4* (the promoter of *IbMYB1-4*) in the anthocyanin synthesis pathway of the PFSP storage roots. In this study, a yeast one-hybrid screening aimed at identifying transcription factors upstream of the promoter *IbMYB1-4* was performed in the storage roots of the purple-fleshed sweet potato. IbPDC, IbERF1, and IbPGP19 were identified as upstream binding proteins for the promoter *IbMYB1-4*. IbERF1 was found to be an upstream transcription factor for the promoter *IbMYB1*, and is implicated in the biosynthesis of anthocyanin in the purple-fleshed sweet potato.

The AP2/ERF families are ethylene response factors (ERFs) involved in multiple physiological processes during the growth of plants, and in their response to biological, as well as nonbiological, stress [6,7]. The endogenous hormone ethylene is involved in growth and development in the roots, leaves, flowers, and fruit [8]. In the last, the endogenous hormone ethylene controls color changes in fruit [9]. ERFs have been reported to regulate color changes in the fruit peel, and anthocyanin biosynthesis, in orange [10], banana and mango [9], pear [11], apple [12], purple tea [13], carrot [14], and hybrid poplar plants [15]. So far, however, ERFs have not been implicated in the biosynthesis of anthocyanin in the storage roots of the PFSP. Instead, IbERF1 has been found to regulate anthocyanin biosynthesis in the PFSP by binding to the *IbMYB1-4* promoter.

## 2. Material and Methods

### 2.1. Plant Material

The PFSP cv. A5 and the white-fleshed sweet potato (WFSP) cv. Yubeibai were cultivated in a garden at South China Normal University, Guangdong, China. Arabidopsis (*Arabidopsis thaliana*) was used for dual luciferase and subcellular localization assays. It was grown at 20 ± 2 °C in a chamber, using a day/night cycle (16 h/8 h), and a constant light intensity (100 μmol/m^2^/s).

### 2.2. Extraction of Genomic DNA and RNA, Gene Isolation, and Sequence Analysis

The root tissue (0.5 g) was ground into a powder with liquid nitrogen using a mortar and pestle. The DNA extraction was then carried out using a plant DNA kit (Tiangen, Beijing, China), and RNA extraction was conducted using a Hipure plant RNA kit (Magen, Guangzhou, China). To eliminate the possibility of DNA contamination, the RNA underwent DNase I digestion using an RNase-free kit (TaKaRa, Shiga, Japan). A BioPhotometer Plus (Eppendorf, Hamburg, Germany) was used to estimate the DNA concentrations and purity by measuring the absorbance at 230, 260, and 280 nm. The DNA samples showed 1.8 ≤ OD_260_/OD_280_ ≤ 1.9 and OD_260_/OD_230_ ≥ 2.0, while the RNA samples showed 1.9 ≤ OD_260_/OD_280_ ≤ 2.0 and OD_260_/OD_230_ ≥ 2.0.

A GoScript^TM^ Reverse Transcription System (Promega, Madison, WI, USA) was used for cDNA synthesis, with the promoter and genomic fragments subsequently cloned and sequenced. Agarose gels (1.2%) were used to analyze the PCR products. The fragments were ligated into plasmid, transformed into *Escherichia coli* DH5α competent cells, then sent to be sequenced by Sangon Biotech, Shanghai, China.

### 2.3. Yeast One-Hybrid (Y1H) Screening

Y1H screening was performed using Matchmaker Gold Y1H Library Screening, as described previously [16]. The RNA was extracted from the storage roots of cv. A5 to build a prey cDNA library, and a cDNA pool was inserted separately into the prey vector pGADT7-Rec. The *IbMYB1-4* promoter was inserted into pAbAi, resulting in pAbAi-bait. The plasmids containing pAbAi-bait were subsequently linearized, and transformed into Y1HGold cells. Colonies growing in a synthetic dextrose medium lacking uracil were then selected. After the minimal inhibitory concentration of aureobasidin A (AbA) for bait strains was identified, the linear pGADT7-Rec vector was co-transformed into bait yeast strains, and then selected using synthetic dextrose (SD)/-Leu/AbA plates. The primers used in the Y1H screening assay are shown in Supplementary Table S1.

### 2.4. Y1H Assay

Y1H was also performed using the Matchmaker Gold Y1H System, as described in another previous study [17]. The Y1H assays were performed to identify the interactions of IbERF1, IbPGP19, and IbPDC with the *IbMYB1-4* promoter. In brief, fragments of the promoter were ligated into the pAbAi vector, while upstream regulators were cloned into pGADT7. The pAbAi was linearized and transformed into Y1HGold cells. The *IbMYB1-4* promoter was inserted into the pAbAi, to create pAbAi-bait. The complete CDSs for IbERF1, IbPGP19, and IbPDC were inserted separately into the pGADT7 vector, to create prey-AD vectors. These were transferred into the bait strain, and cultured on SD/-Leu/AbA plates. The primers used for the Y1H assay are shown in Supplementary Table S1.

### 2.5. Yeast Two-Hybrid (Y2H) Assay

Y2H assays were performed using the Matchmaker Gold Y2H kit (TaKaRa, Dalian, China), as recommended by the manufacturer, but with minor modifications. In brief, the PEG/lithium acetate method was used to simultaneously transform the pGADT7 (AD) and pGBKT7 (BD) vectors into Y2HGold cells. The transcriptional activity for IbERF1, IbPGP19, and IbPDC was investigated using the Y2H assay. The full-length coding sequences for *IbERF1*, *IbPGP19*, and *IbPDC* were cloned into pGBKT7, to construct bait-BD vectors. The PGBKT7-bait and PGADT7-empty vectors were co-transferred into Y2HGold cells. The positive control was pGBKT7-53 co-transformed with pGADT7-53, while the negative control was pGBKT7 co-transformed with pGADT7-53. The primers used in the Y2H assay are shown in Supplementary Table S1.

### 2.6. Dual-Luciferase Assays

Dual-luciferase assays were performed, to quantify the transactivation abilities of IbERF1, IbPGP19, and IbPDC with the *IbMYB1-4* promoter. In brief, the full-length cDNAs for *IbERF1*, *IbPGP19*, and *IbPDC* were inserted into the vector pGreen II 0029 62-SK (Shanghai Qi Ming Biotechnology Co., Ltd., Shanghai, China), while the *IbMYB1-4* promoter was inserted into the vector pGreen II 0800-LUC (Shanghai LMAI Biotechnology Co., Ltd., Shanghai, China). Both these constructs were then transformed into *arabidopsis* protoplasts, as described in a previous publication [18]. The LUC and REN enzyme activity ratio was quantified using the E1910 Dual-Luciferase^®^ Reporter Assay (Promega). Three independent experiments were performed for each of the interactions between the TFs and promoters, and three replicates were used for each experiment. The positive control was a Renilla luciferase gene driven by the 35S promoter in a luciferase vector. Mixtures containing each TF with the empty vector 62-SK were also used on promoters as a control. The primers for the dual-luciferase assay are shown in Supplementary Table S1.

### 2.7. Analysis of Subcellular Localization

For the investigation of subcellular localization, upstream TF CDSs lacking a stop codon were amplified, and cloned into pCambia1300 (Honorgene Co., Ltd. Guangzhou China), with *BamH* Ⅰ and *Hind* Ⅱ. This vector contains the promoter for *UBQ*, as well as the *GFP* gene. Both constructs were transformed into *Arabidopsis* protoplasts, in accordance with a previous publication, while protoplasts were prepared, as described previously, but with slight modifications [18]. The protoplasmic cell culturing time at normal temperature was extended from 20 h to 24 h. The GFP fluorescence was visualized, using confocal microscopy with a Zeiss LSM710 instrument. The primers used in the investigation of subcellular localization are shown in Supplementary Table S1.

### 2.8. Real-Time Quantitative PCR (RT-qPCR)

RT-qPCR was used to evaluate the expression of the upstream TF IbERF1, the TFs IbMYB1, IbbHLH2, and IbWD40, and the structural genes *IbCHI*, *IbCHS*, *IbF3H*, *IbF3′H*, *IbDFR*, *IbANS*, and *IbUF3GT*, in the roots (fibrous, thick, storage) of the PFSP cv. A5, and the WFSP *cv.* Yubeibai. The Prime Script™ RT Master Mix (Takara) was employed, to synthesize the first-strand cDNA from the total RNA, and SYBR^®^ Premix Ex Taq™ II (Takara) was used to perform RT-qPCR. The 20 μL reaction volume contained 10 μL of SYBR^®^ Premix Ex Taq™ II, each primer at 0.5 μM, and 100 ng of template cDNA. The amplification program consisted of one cycle for 10 s at 95 °C; then 40 cycles for 5 s at 95 °C, and 30 s at 60 °C, using a Bio-Rad CFX96 Real-Time PCR system (BIO-RAD, Hercules, CA, USA), as recommended by the manufacturer. The internal control was *IbG14*, and the calculations were performed using the Ct analysis method. The primers for the RT-qPCR experiments are shown in Supplementary Table S1.

### 2.9. Statistical Analyses

Three replicates of each biological sample were evaluated, using one-way analysis of variance (ANOVA). The SPSS 21.0 statistics software package (SPSS Inc., Chicago, IL, USA) was used to determine the significant differences, with Tukey’s honest test (*p* < 0.05). Sigmaplot 12.3 was used to draw the figures.

## 3. Results

### 3.1. Screening of the IbMYB1 Promoter for Upstream TFs

The *IbMYB1* promoter (length 2183 bp) was cut into four segments (−2183 bp~−1684 bp, −1683 bp~−1194 bp, −1193 bp~−694 bp, and −693 bp~−1 bp). A previous study investigated the *IbMYB1-1* promoter for upstream TFs [18]. It was noted that high concentrations of AbA did not suppress the Leu leakiness with *PIbMYB1-2* and *PIbMYB1-3*, with 400 ng/mL being the minimum inhibitory AbA concentration for the PAbAi-*PIbMYB1-4* strains. A total of 519 positive colonies were screened for *PIbMYB1-4*. The Y1H assay identified 130 binding proteins for the *IbMYB1-4* promoter. Supplementary Table S2 shows the analysis of the gene sequences for *PIbMYB1-4*. The IbERF1, IbPGP19, and IbPDC proteins were found to interact with the promoter of *IbMYB1-4*. This gene is involved in the biosynthesis of anthocyanin.

### 3.2. Transcriptional Activities of IbERF1, IbPGP19, and IbPDC

**Figure 1:**
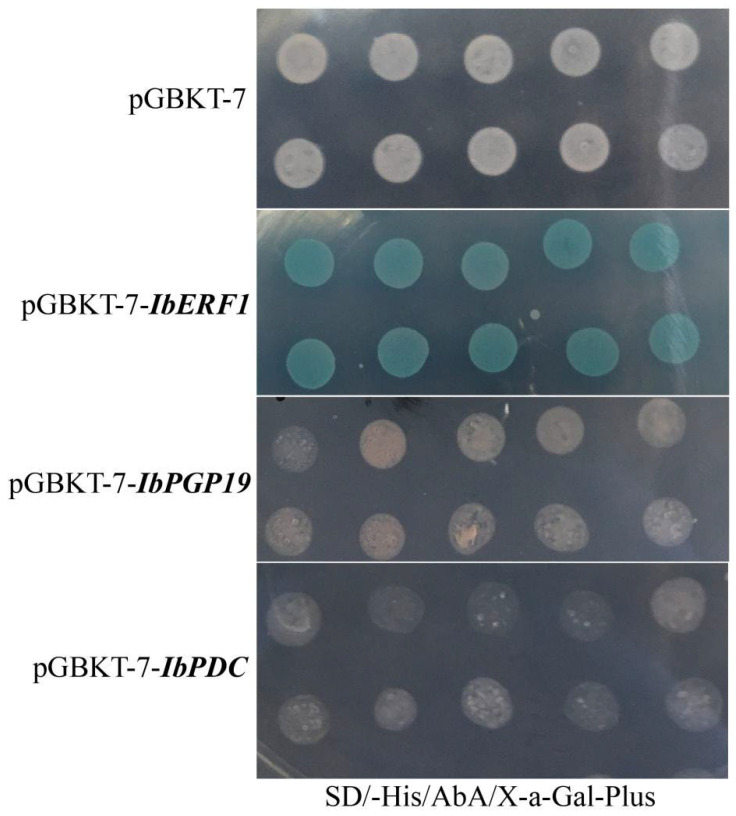
Transcriptional activation of IbERF1, IbPGP19, and IbPDC in yeast cells. The Y2H Gold strains successfully transformed with the corresponding vectors, and grown on SD/-His/AbA/X-α-Gal plates for 3–5 days at 30 °C. The ten points of each treatment were grown on a Petri dish. The growth status of yeast cells evaluated by an X-α-Gal assay was used to monitor transcription activation.

Y2H assays were carried out to determine the transcriptional activities of IbERF1, IbPGP19, and IbPDC. The pGADT7-53+pGBKT7-53 and pGBKT7-*IbERF1/IbPGP19/IbPDC*+pGADT7-empty transformed strains were found to grow on SD/-Trp, SD/-His-AbA plus, and SD/-His-AbA X-a-Gal plus plates, with the color of the emerging yeast colony being blue. The pGADT7-53+PGBKT7 transformed strains were unable to grow on SD/-Trp, SD/-His-AbA plus, and SD/-His-AbA X-a-Gal plus plates (Figure 1). These results suggest that IbERF1 had transcriptional activity.

### 3.3. Interactions of IbERF1, IbPGP19, and IbPDC with PIbMYB1-4

**Figure 2:**
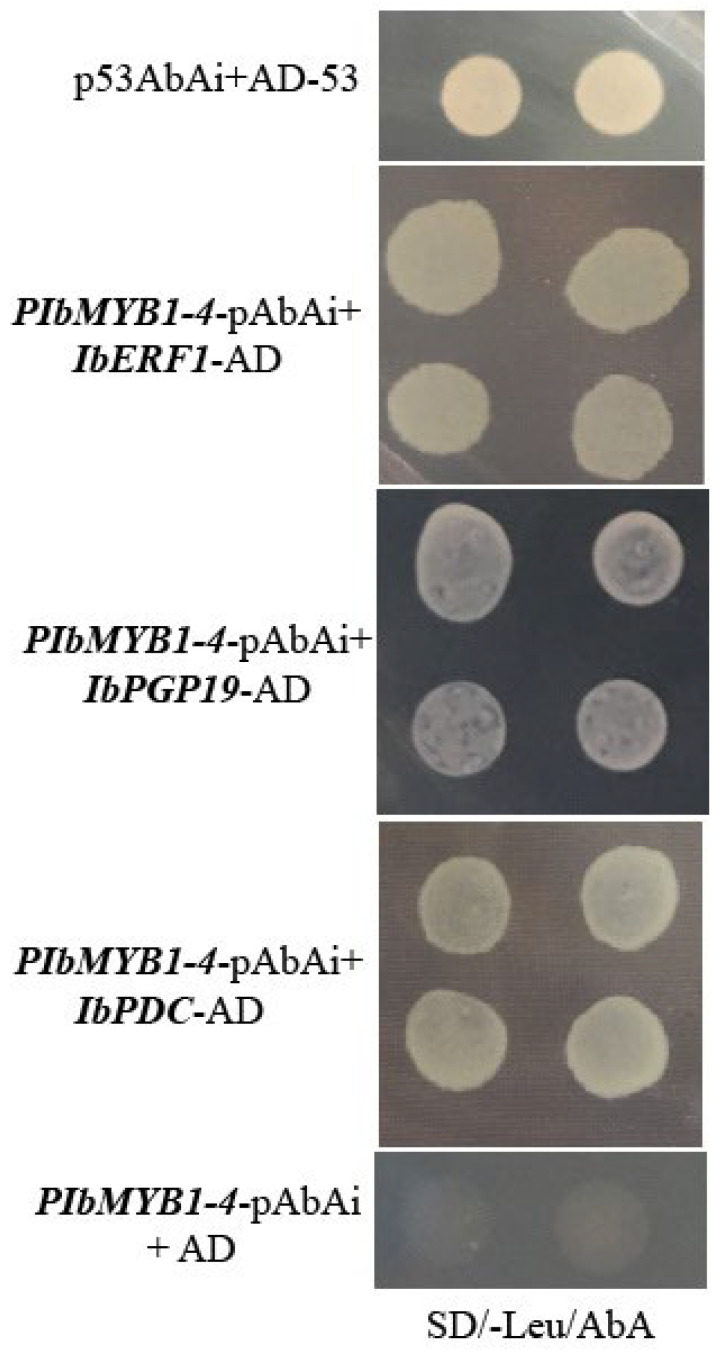
The interaction of IbERF1, IbPGP19, and IbPDC with *PIbMYB1-4*, studied using a yeast one-hybrid assay. The Y1H Gold strains successfully transformed with corresponding vectors, and grown on SD/-Leu/AbA plates for 3–5 days at 30 °C. The four points of each treatment were grown on a Petri dish, and the interactions were confirmed using the yeast cell growth status.

Y1H assays were performed to determine whether IbERF1, IbPGP19, and IbPDC interact with *PIbMYB1-4*. The positive control was found to grow on the SD/-Leu/AbA plates, but not the negative control. All the transformed strains also grew on the SD/-Leu/AbA plates (Figure 2), thereby confirming the interactions of IbERF1, IbPGP19, and IbPDC with *PIbMYB1-4*.

**Figure 3:**
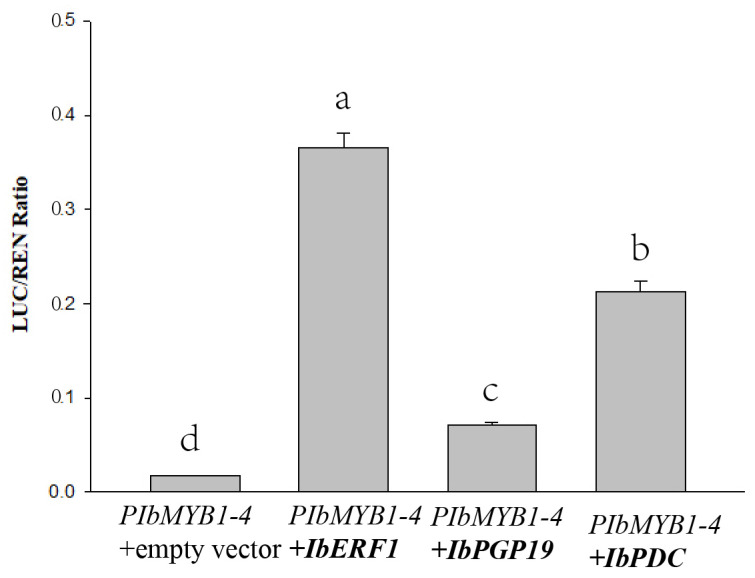
The IbERF1, IbPGP19, and IbPDC proteins activate the *IbMYB1-4* promoters in dual-luciferase assays. The error bars represent the standard deviation (SD). The significance tests are shown as a, b, c, and d. The different lowercase letters in the chart indicate that there is a significant difference (*p* < 0.01).

A dual-luciferase assay was also performed to validate the interactions of IbERF1, IbPGP19, and IbPDC with *PIbMYB1-4*. IbERF1, IbPGP19, and IbPDC each had significant activation effects on *PIbMYB1-4*, as shown in Figure 3. Hence, these results further confirm the interactions of IbERF1, IbPGP19, and IbPDC with *PIbMYB1-4*.

### 3.4. Subcellular Localization of IbERF1, IbPGP19, and IbPDC

**Figure 4:**
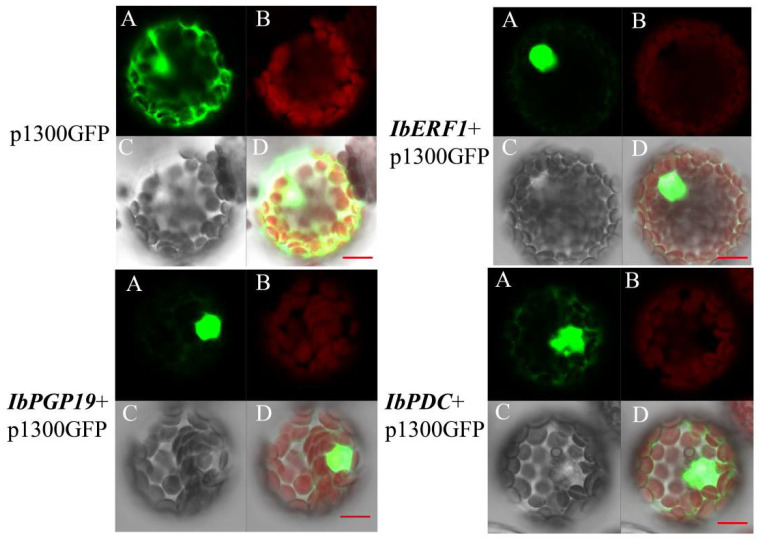
The subcellular localization of the IbERF1, IbPGP19, and IbPDC proteins in *Arabidopsis* protoplasts. The fusion proteins and the GFP control were expressed transiently in *Arabidopsis* protoplasts. The bars represent 20 µm. (**A**) GFP, (**B**) chloroplast, (**C**) light field, (**D**) merged graph.

IbERF1, IbPGP19, and IbPDC were predicted to localize in nuclei. In order to test this, green fluorescent protein (GFP) was fused to the C-terminus of IbERF1, IbPGP19, and IbPDC, and transiently expressed in *Arabidopsis* protoplasts under the control of the UBQ promoter. The GFP control showed green fluorescence in the nucleus and cytoplasm. The IbERF1/IbPGP19/IbPDC-GFP fusions exhibited green fluorescence in the nucleus only (Figure 4), indicating that IbERF1, IbPGP19, and IbPDC are nuclear TFs that regulate the expression of downstream genes.

### 3.5. Expression Characteristics of IbERF1

**Figure 5:**
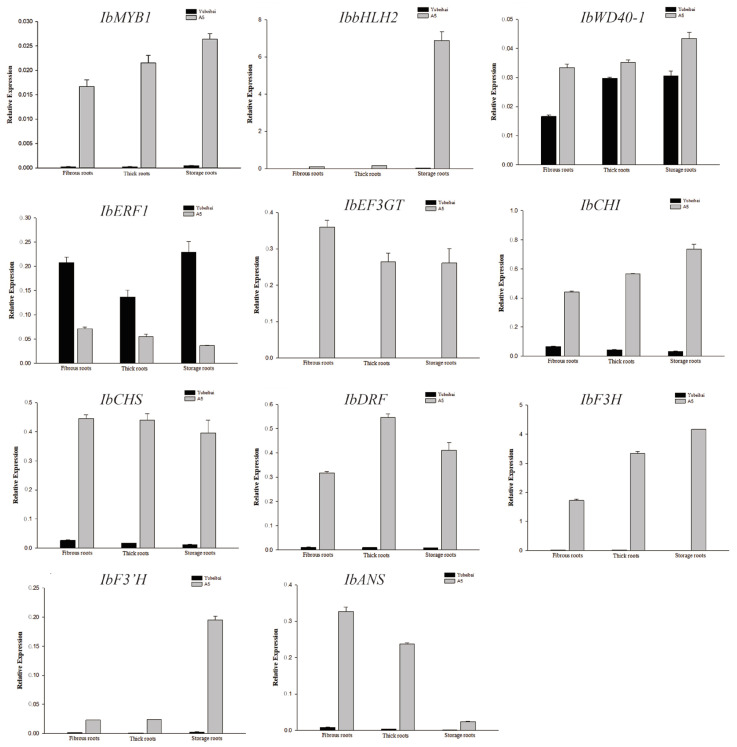
The relative expressions levels of *IbERF1*, *IbMYB1*, and the structural genes involved in the biosynthesis of anthocyanin at the different root stages in the purple- and white-fleshed sweet potato: the fibrous root (diameter < 2 mm), thick root (2 mm < diameter > 5 mm), and storage root (diameter > 5 mm).

Real-time PCR was employed to evaluate the expression of the upstream TFs, TFs, and structural genes involved in the biosynthesis of anthocyanin at the different root stages of the PFSP and WFSP (Figure 5). The expression of the TFs and structural genes at the different root stages of the PFSP was found to be higher than in the WFSP.

## 4. Discussion

Anthocyanin biosynthesis in plants is regulated via the MBW complex. Other TFs, including ERFs, COP1, WRKYs, SPL9, NACs, and DELLA proteins, are also thought to affect the activity of the MBW complex, and hence the biosynthesis of anthocyanin [19,20,21,22,23]. IbMYB1 has previously been implicated in the biosynthesis of anthocyanin in the PFSP [24]. In the present study, the upstream TFs for *PIbMYB1* involved in anthocyanin biosynthesis in the PFSP’s storage roots were screened using a Y1H assay. IbERF1 was found to be an upstream TF for *PIbMYB1-4* in the synthesis of anthocyanin. The interactions between IbERF1 and *PIbMYB1-4* were confirmed using dual-luciferase and Y1H assays. Moreover, IbERF1 was found in the nucleus, using a subcellular localization method. This indicates that IbERF1 is a nuclear protein, and functions as a TF in regulating the expression of downstream genes.

The ethylene response factors AP2/ERFs are a major TF family, involved in multiple physiological processes, including plant growth, and the response to biological and non-biological stress [5,6]. It has been shown previously that TF ERFs regulate the biosynthesis of anthocyanin in plants. In suspension-cultured carrot cells, DcERF1 can upregulate the *DcPAL3* promoter activity, while DcERF2 functions in different ways in anthocyanin biosynthesis [25]. In Salvia miltiorrhiza flowers, the TF AP2/ERFs can regulate anthocyanin biosynthesis, due to the significantly different expression levels between purple and white flowers, as seen in the transcriptome data [26]. TF ERFs have been found to regulate the light- and ethylene-induced biosynthesis of anthocyanin in different species. In plums, seven PsERFs were positively correlated with PsMYB10, and with many structural genes involved in anthocyanin biosynthesis [27]. In *Arabidopsis*, a double mutant (*aterf4* and *aterf8*) reduced the light-induced anthocyanin accumulation, indicating the positive regulation of anthocyanin biosynthesis by AtERF4 and AtERF8 [25]. In pears, TF ERFs regulate the light-induced biosynthesis of anthocyanin [28].

ERF interacts with MYB proteins, and binds to MYB promoters, to regulate the biosynthesis of anthocyanin in plants [24]. In apples, MdERF1B interacts with the MdMYB1, MdMYB9, and MdMYB11 proteins to regulate the biosynthesis of anthocyanin. Furthermore, MdERF1B also binds to the *MdMYB1*, *MdMYB9*, and *MdMYB11* promoters to regulate anthocyanin biosynthesis [14]. MdERF38 binds to the *MdMYB1* promoter to regulate the drought-stress-induced biosynthesis of anthocyanin at the posttranslational level [13]. In pears, PpERF3 interacts with PpbHLH3 and PpMYB114 in the ERF3-MYB114-bHLH3 complex to regulate the red pear color [12], while Pp4ERF24 and Pp12ERF96 regulate the light-induced biosynthesis of anthocyanin [11]. In strawberries, FaERF9 and FaMYB98 combine to form the ERF-MYB complex, and activate the FaQR promoter, thereby increasing the furaneol content in cultivated strawberries [29]. IbERF1 was found here to interact with the *IbMYB1-4* promotor, and thus regulate the biosynthesis of anthocyanin in the PFSP. These results help to clarify the underlying mechanism for anthocyanin biosynthesis, and improve our understanding of the regulatory networks in the PFSP.

## 5. Conclusions

In this research, IbERF1 was identified as a TF for *IbMYB1-4* in the regulation of anthocyanin biosynthesis in the PFSP.

## Data Availability

The data and materials in this manuscript can be accessed.

## Supplementary Materials

The following supporting information can be downloaded at: https://www.mdpi.com/article/10.3390/cimb45070364/s1, Table S1: The primer sets used in this study; Table S2: Information of genes interacted with *pIbMYB1-4* by yeast one hybrid screening.
