# Supplementary material for: Yeast One-Hybrid Screening to Identify Transcription Factors for IbMYB1-4 in the Purple-Fleshed Sweet Potato (Ipomoea batatas [L.] Lam.)
Source: Curr Issues Mol Biol. 2023 Jul 12;45(7):5765–75. doi: 10.3390/cimb45070364 (PMC10378178; doi:10.3390/cimb45070364)
Supplement: Supplementary file 1 [file cimb-45-00364-s001.zip › cimb-2469501-supplementary.pdf]

Supplemental Table S1. The primer sets used in this study

Name	Sequence(5'-3')
**Yeast one-hybrid screening**	
pMYB1-1F	AAATGATGAATTGAAAAGCTTTATTGCTCTCAATGTGCAAGAATCA
pMYB1-1R	GTCGACAGATCCCCGGGTACCTTGATACGCATACCTTATGCCTAA
pMYB1-2F	AAATGATGAATTGAAAAGCTTGTGATTGTTAGTGTTTGGTTGATAGGT
pMYB1-2R	GTCGACAGATCCCCGGGTACCTTGAAAAATCCAGTCAACGAAAAA
pMYB1-3F	AAATGATGAATTGAAAAGCTTGCGCATCCAAACACTGGAAA
pMYB1-3R	GTCGACAGATCCCCGGGTACCACATGATTGTCATTTTATACAAATCGTATT
pMYB1-4F	AAATGATGAATTGAAAAGCTTTTATTACATCAAGCTAAATAAATACGATTTG
pMYB1-4R	GTCGACAGATCCCCGGGTACCTATATATATTGAAGGGTGTCGGAAATTC
pAbAi-F	CAAGTTTGTTTGCTTTTCGTGC
pAbAi-R	GCTGGAATGATGAATAAAGTGCC
**Yeast one-hybrid assay**	
ERF1-ADF	GCCATGGAGGCCAGTGAATTCATGAATTCAGGATTCTCCTCCGA
ERF1-ADR	CAGCTCGAGCTCGATGGATCCTTAAACCAAGGGATTAGGAGTAATTG
PGP19-ADF	GCCATGGAGGCCAGTGAATTCATGGCTTCTCTATGGCCGAAC
PGP19-ADR	CAGCTCGAGCTCGATGGATCCCTAAGAATTGAATTTACAACTTGCTGTT
PDC-ADF	GCCATGGAGGCCAGTGAATTCATGGACACGAAGATTGGATCCA
PDC-ADR	CAGCTCGAGCTCGATGGATCCTTACTGTGGGTTTGGTGGCC
pGADT7-F	CGATGATGAAGATACCCCACCAAACC
pGADT7-R	ACTTGCGGGGTTTTTCAGTATCTACG
**Yeast two-hybrid (Y2H) assay**	
ERF1-BDF	ATGGCCATGGAGGCCGAATTCATGAATTCAGGATTCTCCTCCGA
ERF1-BDR	CCGCTGCAGGTCGACGGATCCTTAAACCAAGGGATTAGGAGTAATTG
PGP19-BDF	ATGGCCATGGAGGCCGAATTCATGGCTTCTCTATGGCCGAAC
PGP19-BDR	CCGCTGCAGGTCGACGGATCCCTAAGAATTGAATTTACAACTTGCTGTT
PDC-BDF	ATGGCCATGGAGGCCGAATTCATGGACACGAAGATTGGATCCA
PDC-BDR	CCGCTGCAGGTCGACGGATCCTTACTGTGGGTTTGGTGGCC
pGBKT7-F	TAATACGACTCACTATAGGGCGA
pGBKT7-R	GACTCTTAGGTTTTAAAACGAAAA
**Dual-luciferase assay**	
pM1-4-0800F	CTATAGGGCGAATTGGGTACCTTATTACATCAAGCTAAATAAATACGATTTG
pM1-4-0800R	TGTTTTTGGCGTCTTCCATGGTATATATATTGAAGGGTGTCGGAAATTC
p0800-LUC-F	CAAGGCGATTAAGTTGGGTAAC

p0800-LUC-R	CTCTCCAGCGGTTCCATCTTCC
ERF1-62-SKF	AGGACAGCCCAAGCTGAGCTCATGAATTCAGGATTCTCCTCCGA
ERF1-62-SKR	GGTACCGGGCCCCCCTCGAGTTAAACCAAGGGATTAGGAGTAATTG
PGP19-62-SKF	AGGACAGCCCAAGCTGAGCTCATGGCTTCTCTATGGCCGAAC
PGP19-62-SKR	GGTACCGGGCCCCCCTCGAGCTAAGAATTGAATTTACAACTTGCTGTT
PDC-62-SKF	AGGACAGCCCAAGCTGAGCTCATGGACACGAAGATTGGATCCA
PDC-0800R	GGTACCGGGCCCCCCTCGAGTTACTGTGGGTTTGGTGGCC
p62-SK-F	CATATAAGGAAGTCATTTCATT
p62-SK-R	TCCCTTATCGGGAAACTACTCA
**Subcellular localization analysis**	
ERF1-1300F	TTTTCTGATTAACAGGGATCCATGAATTCAGGATTCTCCTCCGA
ERF1-1300R	GCTCTCGAGACTAGTGGTACCAACCAAGGGATTAGGAGTAATTGATG
PGP19-1300F	TTTTCTGATTAACAGGGATCCATGGCTTCTCTATGGCCGAAC
PGP19-1300R	GCTCTCGAGACTAGTGGTACCAGAATTGAATTTACAACTTGCTGTTCT
PDC-1300F	TTTTCTGATTAACAGGGATCCATGGACACGAAGATTGGATCCA
PDC-1300R	GCTCTCGAGACTAGTGGTACCCTGTGGGTTTGGTGGCCTG
pCambia1300-F	TACTCTTCGATTTGTGATTTC
pCambia1300-R	CATCGCAAGACCGGCAACAGG
**Real-time quantitative PCR**	
Q-G14-F	GTGCGGAAACTGCGACTGC
Q-G14-R	CGTCCATCTTGCTTCCCTTCCT
Q-MYB1-F	ATGGTTATTTCATCTGTATGGTCGG
Q-MYB1-R	CTCAAAAGTTGGTCTTCTTCTTCG
Q-ERF1-F	AACGGGATAAGGGTTTGGCTGGG
Q-ERF1-R	CGGACAGTCTCCACGGGGAAGTT
Q-CHS-F	AAAACCTGAAAAACTCCGAGCCACG
Q-CHS-R	CAAACCCAAACAGCACACCCCAC
Q-CHI-F	GAAGGTGTCGGAAAACTGCGTTG
Q-CHI-R	GGGGTGATTGAGTGAAGAAAATAGAGG
Q-F3H-F	TTCATCGTTTCCAGCCATCTCCA
Q-F3H-R	TTTTCCGTTACTGCCCTCCACCC
Q-F3’H-F	CTGTGGAGTGGGCATTCGCAGAA
Q-F3’H-R	AGAGAGTGGGGTGGATGGGTGTAGC
Q-ANS-F	AATAATGCTAGTGGGCAGCTTGAGTG
Q-ANS-R	AAGGAGTTTTAGGCCAAATGGAGAGA
Q-DFR-F	CTGCTGGCTTTATCGGCTCCTGGT
Q-DFR-R	TGTCAAATTCGTGTCCGCTTTCG

Q-UF3GT-F	GCGTTCCTTGGATTTCTTTTTGG
Q-UF3GT-R	TGACATTCCTGGGATTACTTTCAGCT

Supplemental Table S2. Information of genes interacted with *pIbMYB1-4* by yeast one hybrid screening

NO	Gene	Accession	Length(bp)	NO	Gene	Accession	Length(bp)
1	*PLP-3A*	AT3G50960.1	1732	66	*TRM140A*	AT2G26200.1	3982
2	*IPCS1-3*	AT3G54020.1	2639	67	*UBQ3*	AT5G03240.1	1854
3	*EMB60*	AT1G01040	6276	68	*ER1*	AT5G09410.3	6594
4	*RHM1-2*	AT1G78570.1	2899	69	*NRP1*	AT1G74560.3	2466
5	*AIR12*	AT3G07390.1	1196	70	*GSR5*	AT5G37600.1	2600
6	*VPS2*	AT2G06530.1	2009	71	*ATG3*	AT5G61500.1	3092
7	*SORF1*	AT1G10682.1	702	72	*LYR*	AT5G51960.1	1092
8	*GER5*	AT5G13200.1	1506	**73**	***ERF1***	**AT3G23240.1**	**988**
9	*HIS4*	AT2G28740	312	74	*CBR1*	ATCBR	2186
10	*HAT22*	AT4G37790.1	1635	75	*MKK4*	AT1G51660.1	2016
11	*VHA-F*	AT4G02620.1	2108	76	*RTNLB5*	AT4G23630.1	3039
12	*RAN*	AT5G20010.1	2237	77	*TFIIS*	AT2G32820.1	1163
13	*RPS5B*	AT2G37270.1	1795	78	*RABA4A*	AT5G65270.1	1617
14	*CNX1*	AT5G61790.1	2838	79	*CID6*	AT5G25540.1	1562
15	*PFD5*	AT5G23290.1	1563	80	*U2AF65B*	AT1G60900.1	4160
16	*EDA16*	AT1G61140.1	6270	81	*SNX2A*	AT5G58440.1	3164
17	*TRM9*	AT4G00770.1	2321	82	*TPS7*	AT1G06410.1	4150
18	*XTH*	AT1G10550.1	933	83	*RP*	AT5G16130.1	1779
19	*PS2*	AT1G73010.1	1821	84	*CAR6*	AT1G70800.1	1434
20	*NDK1*	AT4G09320.1	883	85	*KHZ2*	AT5G06770.1	2299
21	*OXS3*	AT5G56550.1	1119	86	*Hsp 70*	AT3G12580.1	2770
22	*PML3*	AT5G36290.1	2521	87	*IMI*	AT5G62350.1	1234
23	*TRAB*	AT5G45130.1	2280	88	*GSM1*	AT5G23010.1	3805
24	*AS*	AT2G21390.1	4967	89	*eIF3*	AT5G37475.1	2719
25	***PDC1***	**AT4G33070.1**	**2547**	**90**	*GF14*	AT1G78300.1	2250
26	*ARM*	AT3G20170.1	1824	91	*PSI1*	AT1G34320.1	4319
27	*Zinc*	AT5G25830.1	1706	92	*LRTPK*	AT4G29990.1	4347

28	*TRAF*	AT3G61790.1	2137	93	*AS2*	AT1G65620.1	2834
29	*TSS*	AT1G68490.1	2802	94	*PS2*	AT1G73010.1	1821
30	*MUM4*	AT1G53500.1	2814	95	*RRN18*	ATMG01390.1	1935
31	*APG1*	AT3G63410.1	1531	96	*LOG3*	AT2G37210.2	2948
32	*MBOAT*	AT1G57600.1	4711	97	*RD2*	AT2G21620.2	1684
33	*WIP*	AT1G08290.1	3382	98	*CDPK9*	AT5G23580.1	4420
34	*SNF7*	AT2G06530.1	2009	99	*PML3*	AT5G36290.1	2521
35	*OGH17*	AT5G55180.2	1933	100	*HRQ1*	AT5G08110.1	5412
36	*DAT*	AT2G03800.1	2438	101	*HDH1*	AT4G20930.1	2856
37	*SAP13*	AT3G57480.1	2102	102	*50A*	AT1G36980.1	2318
38	*MEB2*	AT5G24290.1	2617	103	*ATP6*	ATMG00410.1	1321
39	*GRP4*	AT3G23830.1	1869	104	*LEA*	AT5G53730.1	1382
40	*RAB8Rab*	AT3G53610.1	2561	105	*PG*	AT2G05171.1	958
41	*NCL-like*	AT1G53210.1	3407	106	*ODB1*	AT1G71310.2	1599
42	*V-ATP*	AT4G02620.1	2108	107	*ARA7*	AT4G19640.1	2551
43	*HERK*	AT3G46290.1	3386	108	*LNK1*	AT5G64170.2	4358
44	*WRKY7*	AT4G24240.1	2236	109	*CD*	AT2G35795.1	1401
45	*RSZ22*	AT4G31580.1	2492	110	*MPK8*	AT1G18150.2	3447
46	*RXF*	AT1G58430.1	1610	111	*MUG3*	AT1G06740.1	2638
47	*SAP*	AT5G66840.1	2683	112	*AHS*	AT3G23570.1	2048
48	*APA1*	AT1G11910.1	4318	113	*OPS*	AT3G09070.1	2735
49	*EMB2296*	AT2G18020.1	1410	114	*ESD4*	AT4G15880.1	3610
50	*IF2/IF5*	AT1G77840.1	2365	115	*α1-COP*	AT2G21390.1	4967
51	*RAN3*	AT5G55190.1	3333	116	*MPK18*	AT1G53510.1	4060
52	*NKS1*	AT4G30996.1	1724	117	*EF1B*	AT1G57720.1	3060
53	*SNF2*	AT1G77840.1	2365	118	*HAT14*	AT5G06710.1	2608
54	*RPS5B*	AT2G37270.1	1795	119	*BICE1*	AT4G05440.1	1423
55	*A20/AN1*	AT1G51200.1	2354	120	*TRNS.1*	ATMG00230.1	87
56	*IF6*	AT3G55620.1	2499	121	*EIF6A*	AT3G55620.1	2499
57	*PH1*	AT5G05710.1	770	122	*ARA6*	AT3G54840.1	2695
58	*ABCI12*	AT3G21580.1	2078	123	*HMGB3*	AT1G20696.2	2047
59	*CNX1*	AT5G61790.1	2838	124	*SNX2B*	AT5G07120.1	2663
60	*GLR5*	AT1G05200.1	3967	125	*EP70*	AT5G37310.1	3802
61	*PFD5*	AT5G23290.1	1563	126	***PGP19***	**AT3G28860.1**	**7574**
62	*TIF3K1*	AT4G33250.1	2158	127	*LHP1*	AT5G17690.2	5669
63	*PRR8*	AT5G17640.1	2382	128	*PT2*	AT5G28143.1	894
64	*ASG1*	AT5G17640.1	2382	129	*ASP1*	AT2G30970.1	3222
65	*XTH27*	AT2G01850.1	2308	130	*BPM5*	AT5G21010.1	2751

pIbMYB1

TATTGCTCTCAATGTGCAAGAATCAAATGAAGTATCAATCAGAAGTTTAATTGG
GACATATTTATTGGTAGCAAATAAAAACAAACTTGTTAACTCTATTACTAAGTC
AAAAACCTTGTGGTCTAGTGACACCCGATTGCACCCCCACATGGGAAGGGTGA
GTTCGAGCTTCAGTAAATGTGATATTGGCTCTATGTGCTTCATTTGGTTGAGAA
AATAGCTATAAACATATACTACATTGTAATGGAGTCAATAGTTCTCAAAAAAA
AAAAAAAAGTCTTCGACCGTTTGCCTACTAACCTAGTATTAATGGGAGTTTCTC
TCATACATTTTATAAAAATTTAAAAAGAGTAATGCTATTTCTCTCTAAAAAAAA
TTCTTCTCAAAATTTCCCGTAACATTATTTGATTGGCCACTTTTCTTTTTCATTAA
GGGTCCATCTGAAAAACAGGAAGGATGTGTTTGGTTGGGGGGTTTAGGCATAA
GGTATGCGTATCAAAGTGATTGTTAGTGTTTGGTTGATAGGTTTTGTGAATGTT
ACTATGGGTTTGGAATACCCCATTAATGGCAAAACCCATACCCTTATTAAATAA
GGGTTTCATCTCCCTTCATCATTTTTTCCCCAATTATTAATAACCATTCCCATTC
CACCCAACTACCAAACATGCTAAATACTTCCACCAAAACCCATTACCCTTACCA
AGTATTTGATACCCATTCCGATTCCGATTCCGATTCCCACGTGCGAACCAAACG
CACCCGAAAATGTCTTCTGAAAAATGAGTACAATTGTTTAATTAAACATTTTAA
TTATTTTATTTAAAATATAAAAAATTATTTATAATAATATTAAAAATATGTTATT
TATTATTGTTATTATTATTTTTTAAATGATGGTTTCCGACGGAATGGTTTCTGCC
GAAAACGAGAGCCTAGGTCCAGAAAATGTAAAGATTTTCCCAGTCAATGGAAA
GTGTTTTTCGTTGACTGGATTTTTCAAGCGCATCCAAACACTGGAAACTCCGAA
AATGATTTTCAGAAAACATTTTTCGAGTTTTCAAACACCCTAAATGTGTGTTGG
TGTGTAGTCAGTTAATTCATTGCACCCAATGATTATAAAACATGTCATGCAGAG
AATTTAAAGAGAGAAAAAATTAGAATGAGTAAGAAGCATTTTTCAATAAATAA
TTAAATAAATAAAAGATTCCTATGTTATGACAAATTTTTGGGCAATTAAGATAT
TGCTTAAATTATATAATTTTTACACAATATTATAATACTCATCCAACGGTTCTCG
ATAGAGGCATACAAGTCGTGCTCTAGATATTTAGAAATATTTGGAGCAAATCC
AATGATTTGACACAGCAAATATTTGTGTGGCCCACAAAATTTTTTTATGCACCT
CAAAAATTTAATGATGTCTAATATAATGCATTAGTTAATTTCTTACTTATTACAT
CAAGTTAAATTAATACGATTTGTATAAAATGACAATCATGTTTATTACATCAAG
CTAAATAAATACGATTTGTATTAAATTAATACAATAATAATAATAATCAAGGAC
AATTTAGTCATTTTCTTTGTTTGTTCTTTTTTCAAGATGCATTAAATTCTAATTTC
TAGGAGATATATGAATTGCAATTTCACAAATAGAAAAAATTGCAATTCCATCA
ATTCAAACACCGTAGTTTATAGCTCCGTTAAATTGCATTGTAATTGAATTAAAA
TTTATGTCAACCAATTAACCGAACACCCTCTAAGGGAATCTCTTGTAATCTAAA
AAAAAATGAATTATCAAAAATTTAAATGTTGTTTGTAAACCTGTCTTATTCACA

AACTTTATGTGATCATACAGAATCTACATAATGATTTTAATAAAAAAAAAAAA
AGTTAAGAAAACAAGTGTATTTCGAAAAAAAAAAAAAAAACAAGTGGAAAAC
ATGTGCAGTGTCATCATGTAAGTACTCAGTGGTATATATAGTAGCTGTGCTAAC
TATATTGCAGGGCATACTTATACCAATAATTGGATGCTGCGCTATCTTCTATTAT
ATTACTCAAGGTCGTTTCTCCATCTTTTCTTCACTTTTTTTTTCCGGAATTTTGGT
GCTACCACACCCAAGTAGCCTACCTATACTACAACAACCTTAGCTAAGAATTTC
CGACACCCTTCAATATATATA

IbERF1

ATGAATTCAGGATTCTCCTCCGATTCATCGCCGGAATATTTCCCGTGGGATTGC
CGGAATTTCGACAGCTCCCTCCCGTTCAACGTTGATGACTCCGAAGAAATGCTC
CTCTTCGGGGTTCTGTCTCAGGCGGCGGCGCGAGAGAACTCCGAGACCAATTCC
TCGGATTACAGCGTGAAGGAAGAGGAGGTGACCTCGGAGACTCCGAAGAAGG
AGCAGCGGTCGTTCCGCGGCGTGAGGCGGCGGCCCTGGGGGAAATTCGCGGCG
GAGATAAGGGATTCGACGAGGAACGGGATAAGGGTTTGGCTGGGAACCTTCGA
CAAAGCAGAGGAGGCGGCTCTGGCCTACGACCAAGCGGCGTTCTCGATGCGGG
GGCCCATGGCGGTCTTGAACTTCCCCGTGGAGACTGTCCGGGAATCTCTCCGGG
AAATGAGGTGCCCCGTCGAGGAGGGCGGCTCTCCCGTGGTGGCGCTCAAGCGG
AGACACTCCTTGCGGCAGCGGGGATTGAACCGGAGGAGCGGCAAACGGCGGG
AGGTGAAGGTGGAGAATGTGGTGGTGTTTGAGGACTTGGGTGTTGATTACTTG
GAACAACTTTTGAGTTCAAGTGGGACTACAACAACATCATCATCATCATCAATT
ACTCCTAATCCCTTGGTTTAA

IbPGP19

ATGGCTTCTCTATGGCCGAACAACCCAATCGCACACAATATACACCAAATCAAATCCACAA
CCCAAAAACCCTACCATCAGTTCGTACATTTTCACTTCGATTCTAACCCTAAATACTTGATA
CCTAACTGTTTCTGCAAATCTATGCTCCCACAATCCTCAAAAATACCTTACATGCCCGATTA
TTCAAATCTTTATAAGTAATCGTTTGAATAACCATCAACGATTACAAATCAACAAACCCTAA
CCAACAAATCTACGAAATCAAATACAACAAATCACAGATACGAAAACCATCGGAGAAGAA
AGAAGAATGGCGAGCACAAGTTATACGAAGTACAATAGCCCACAGATAGGACACCTAAAT
GAGAAGCAATTAGTGAAAAGGTTGGACATTAATGAAAAGGTAGAATGGCAGATTATTTCA
ATACAAAAAAAAAAAAAAAAGCTTCCTCCTCCTCCATCTTTCTGTAAACCTCCATTTCTTGA
ACATACTGTTCCTTGTTCTTTGCTGCAATCTGTATCATGCAAAAGAGGCTCATGTGTCACGG
TATTTTAGAGCAATACTAAAAATGGGGGATTTAAGAAACTGTCATCCTGCACATAAATGTT
AGCAAGCACCTCAGTAGATGCAAGAATTGTTGAAGCTAAATATTGCATCTGTATAAACAGG
AAACTCCTTGCAAGAGTGGCTGAAAAACCTGGCCAGACATGCTGGAACTTGAAGGAAGCTA
AGAGAACAGCAAGTTGTAAATTCAATTCTTAG

IbPDC

ATGGACACGAAGATTGGATCCATTGACACGTGTAAGCCATTGAACAATGACGTAGGCAACG
CCCCCTCCACCGGCGCCTTCACCATCCAGAACTCCTCCTTCCACTGCAGCTCCGCCGAGGCC
ACGCTCGGCCGCCACCTGGCTCGCCGCCTCGTGCAGATAGGGGTGAGTGACGTCTTCGCCG
TCCCCGGCGACTTCAACCTCACGCTCCTCGACCATCTCATTGACGAGCCCGGGCTCAACCTC
ATCGGCTGCTGCAACGAGCTCAATGCCGGATACGCCGCCGACGGTTACGCTAGGTCCCGCG
GAGTCGGTGCTTGCGCCGTCACCTTCACCGTCGGAGGCCTCAGCGTCCTCAATGCCATTGCC
GGCGCCTACAGCGAGAACCTCCCTCTCATATGCATTGTTGGCGGCCCCAACTCCAATGACTA
CGGGACCAACCGGATCCTCCATCACACCATCGGCTTACCCGATTTCACTCAGGAGCTTCGTT
GCTTTCAAACCGTCACTTGTTATCAGGCCGTGGTGAACAATTTGGAGGATGCGCATGAGTTG
ATCGATACTGCGATATCAACGTCGTTGAAAGAGAGTAAGCCGGTTTACATAAGCATAAGCT
GTAACCTGGCAGGGATTCCACATCCTACCTTCAGCCGTGAACCGGTTCCATTTTCGATCAAT
CCACGATTGAGTAATCAGATGGGGTTGGAAGCGGCGGTGGAAGCGGCGGCAGAGTTTCTG
AACAAGGCTGTAAAGCCAGTTATGGTGGGAGGTCCAAAAATGCGCGTTGCAAAAGCCTGC
AAATCCTTCGTGGAGTTGGCGGATGCTTGTGGCTATGCCGTGGCGGTTATGCCGTCGGCCAA
GGGGCTAGTTCCCGAACACCACCCTCATTTCATCGGAACTTACTGGGGCGCCGTTAGCACGT
CGTTCTGCGCTGAGATCGTGGAATCCGCCGATGCGTACTTGTTCGCCGGACCTATATTCAAC
GACTACAGCTCCGTCGGCTACTCCCTGCTTCTGAAGAAGGAGAAGGCGATCATCGTTCAGC
CTGATCGCGTGACGATCGGAAACGGACCTGCATTTGGTTGTGTCCTGATGAAGGATTTCCTA
ACCGCTTTGGCGAAGAGACTCAACTGCAACAAGACTGCTTACGAAAATTACTACAGGATTT
ATGTTCCTGAAGGGAGTCCTGTCAAATGTGAGCCTAAAGAGCCATTAAGGGTGAATGTTCT
CTTCCAGCACGTTCAGAAAATGCTGTCCGGCGAAACCGCCGTGATTGCTGAGACAGGGGAT
TCCTGGTTCAATTGCCAGAAGCTGAAACTACCAGAAGGGTGCGGGTATGAGTTCCAAATGC
AGTATGGCTCAATTGGTTGGTCTGTTGGTTCCACTCTTGGCTATGCTCAAGCTGTTCCCGAG
AAGCGGGTGATTTCCTTCATCGGCGACGGTAGCTTTCAGGTGACCGCTCAGGATGTGTCGA
CGATGCTGCGATGCGGGCAGAGGAGCATAATCTTCCTCATCAACAATGGTGGCTACACAAT
CGAAGTTGAAATCCATGATGGACCTTACAACGTAATCAAGAACTGGAATTACACTGCCCTT
GTTGATGCAATCCACAACGGCCAGGGCAAATGCTGGACAACCAAGGTTCGTTACGAAGATG
AGTTAGTTGCAGCAATCGAGACAGCGACAGGAGCAAAGAAAGATTGCCTATGCTTTATTGA
AGTAATTGCTCACAAGGATGACACGAGCAAAGAATTGCTGGAATGGGGGTCGAGGGTCTCT
TCAGCCAACAGCAGGCCACCAAACCCACAGTAA
